# A systematic review of team-building interventions in non-acute healthcare settings

**DOI:** 10.1186/s12913-018-2961-9

**Published:** 2018-03-01

**Authors:** Christopher J. Miller, Bo Kim, Allie Silverman, Mark S. Bauer

**Affiliations:** 1 Center for Healthcare Organization and Implementation Research (CHOIR), VA Boston Healthcare System (152M), 150 South Huntington Avenue, Boston, MA 02130 USA; 2 Harvard Medical School, Department of Psychiatry, Boston, USA

**Keywords:** Teamwork, Team training, Team-building intervention, Non-acute

## Abstract

**Background:**

Healthcare is increasingly delivered in a team-based format emphasizing interdisciplinary coordination. While recent reviews have investigated team-building interventions primarily in acute healthcare settings (e.g. emergency or surgery departments), we aimed to systematically review the evidence base for team-building interventions in non-acute settings (e.g. primary care or rehabilitation clinics).

**Methods:**

We conducted a systematic review in PubMed and Embase to identify team-building interventions, and conducted follow-up literature searches to identify articles describing empirical studies of those interventions. This process identified 14 team-building interventions for non-acute healthcare settings, and 25 manuscripts describing empirical studies of these interventions. We evaluated outcomes in four domains: trainee evaluations, teamwork attitudes/knowledge, team functioning, and patient impact.

**Results:**

Trainee evaluations for team-building interventions were generally positive, but only one study associated team-building with statistically significant improvement in teamwork attitudes/knowledge. Similarly mixed results emerged for team functioning and patient impact.

**Conclusions:**

The evidence base for healthcare team-building interventions in non-acute healthcare settings is much less developed than the parallel literature for short-term team function in acute care settings. Only one intervention we identified has been tested in multiple non-acute settings by distinct research teams. Positive findings regarding the utility of team-building interventions are tempered by a lack of control conditions, inconsistency in outcome measures, and high probability of bias. Considering these results alongside the well-recognized costs of poor healthcare teamwork suggests that additional research is sorely needed to develop the evidence base for team-building in non-acute settings.

## Background

Healthcare delivery is increasingly based on healthcare teams, with an emphasis on coordination among providers from different disciplines [1, 2]. Good team functioning is associated with improved patient outcomes, heightened staff satisfaction, and reduced burnout [3–5]. In contrast, poor team functioning is associated with poor patient care through adverse events, lack of coordination, and spiraling costs [6–8].

Despite this, many healthcare providers have not received adequate training in team-based approaches to healthcare [9]. This has led to recent calls for more emphasis on teamwork in medical education [10]. In addition, a variety of models, guidelines, and trainings have been developed to support development of effective healthcare teams in hospitals and other clinical settings. Specifically, numerous trainings are meant to improve team functioning in emergency settings, acute care wards, and surgery departments (for example see recent reviews [11, 12]). Many of these team-building approaches are based, directly or indirectly, on the aviation-derived principles of crew resource management or crisis resource management (CRM [13]). They are therefore typically designed to prepare providers for medical emergencies that can develop and escalate rapidly (e.g. cardiac arrest or unexpected surgical complications), with an emphasis on in-the-moment situation monitoring and communication.

In contrast, there are relatively few interventions to enhance healthcare teamwork for non-acute or ambulatory care settings, where teamwork challenges may unfold over days, weeks, months, or even years rather than seconds or minutes. Given that the long-term treatment of chronic disease represents an increasing burden on healthcare systems [14–16], this relative shortage of team trainings for non-acute settings represents an important gap to be addressed [11].

### Purpose of the study

Given this state of affairs, we had three goals for this review. First, we aimed to describe the characteristics of team-building interventions that have been applied in non-acute healthcare settings. Second, we aimed to identify the characteristics of empirical studies that have tested these team-building interventions in such settings. Third, we aimed to evaluate empirical results of these team-building interventions in four outcome domains: trainee evaluations, teamwork attitudes/knowledge, team functioning, and patient impact. To our knowledge, this is the first review of team-building interventions to focus specifically on non-acute settings.

### Definitions

For this review we have adopted the definition of team-based healthcare put forth by Mitchell and colleagues in their Institute of Medicine (IOM) discussion paper [1], itself adapted from Naylor and colleagues [17]:

“Team-based health care is the provision of health services to individuals, families, and/or their communities by at least two health providers who work collaboratively with patients and their caregivers—to the extent preferred by each patient—to accomplish shared goals within and across settings to achieve coordinated, high-quality care.” [1] (page 5).

Furthermore, there is diversity in the literature regarding how to label team-building approaches themselves, with some authors using the term “team-building intervention” (e.g. [18]), while others use some variation of “team training” (e.g. [11]), some combination of the two (e.g. [19]), or one of a host of other terms (e.g. [20]). For simplicity we have chosen to adopt the term “team-building intervention” to refer to any systematic approach to improving healthcare team functioning for the purposes of this review (see Methods for details).

### Guiding conceptual model

**Fig. 1:**
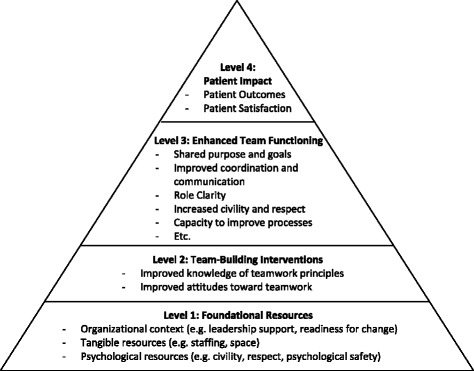
Team Effectiveness Pyramid (a conceptual model for non-acute healthcare team-building)

We developed a guiding conceptual model of non-acute healthcare team-building based on previous literature (Fig. 1, which we have entitled the Team Effectiveness Pyramid). We propose as a starting point that building effective healthcare teams in non-acute settings requires a baseline level of resources (Pyramid Level 1), including a supportive organizational context [5], basic tangible resources such as staffing [3, 21] and space [22], and psychological resources in the form of civility, mutual respect, and psychological safety [23, 24] for the staff who comprise the team. The model proposes that these preconditions provide fertile ground for team-building interventions (Pyramid Level 2) to lead to enhanced teamwork (Pyramid Level 3). The bullet points at this level are not meant to be comprehensive, but rather to list some of the qualities frequently cited in this domain [5]. Finally, our model posits that good teamwork will in turn lead to improved patient impact in the form of both clinical outcomes and patient satisfaction (Pyramid Level 4) [1, 2].

The four outcomes we chose to investigate for this review align closely with the Team Effectiveness Pyramid. Specifically, as described above, our outcome domains included trainee evaluations (Pyramid Level 2), teamwork attitudes/knowledge (Pyramid Level 2), team functioning (Pyramid Level 3), and patient impact (Pyramid Level 4). While we believe that foundational resources (Pyramid Level 1) are crucial to healthcare team-building, addressing this issue was beyond the scope of this review, as most studies of healthcare team-building provide only general information about the settings in which they are conducted.

## Methods

We searched two electronic databases (PubMed and Embase) for English-language manuscripts from the earliest available date in each database through March of 2017. Our first goal was to identify reviews of team-building interventions (Review Stage 1). We then used those reviews to identify articles describing team-building interventions for non-acute care settings (Review Stage 2). Finally, we conducted follow-up literature searches to identify articles describing studies of those interventions (Review Stage 3). This multi-step search process (starting with a review of reviews) provides a broad initial view of the literature, and has been used in at least one previous review of team trainings in different contexts [25].

### Identifying reviews (review stage 1)

Our initial search terms consisted of the following: ((“Patient Care Team”[Mesh]) AND (model[All Fields] AND Review[ptyp])); ((“team training”[tiab] OR “teamwork training”[tiab]) AND review[tiab]); ((“Patient Care Team”[Mesh] OR “patient care team” OR team*[tiab] OR interdisc*[tiab] OR multidisc*[tiab]) AND (model[tiab] OR framework[tiab]) AND review[tiab]). The first author screened all titles resulting from these searches to identify potentially relevant papers for full-text review. Inclusion criteria for these reviews consisted of the following:

- A focus on healthcare teamwork as described above.
- Inclusion of at least one team-building intervention that is explicitly meant to be applied in non-acute healthcare settings. These most commonly include outpatient or ambulatory care clinics, but could also include inpatient settings if the focus was on teamwork required over the course of a patient’s stay (and not just teamwork needed for emergencies).
- Application of systematic rigor (e.g. systematically review the literature, establish statistical methods for evaluating outcomes across studies), although we ultimately relaxed this criterion to maximize our ability to identify trainings that had not yet been exhaustively tested and published.

### Identifying team-building interventions (review stage 2)

We read the manuscript body and reference list of each of the reviews identified in Review Stage 1 above, with a goal of identifying team-building interventions. Inclusion criteria at this stage consisted of the following:

- Inclusion of domains or elements to pursue in improving teamwork within a (healthcare) team. Interventions focusing solely on improving clinical care processes (such as the adoption of evidence-based practices) or delineating team structure or roles (such as the Collaborative Care Model or CCM [26]) were not included unless they also included a specific focus on improving teamwork.
- A focus on the team level—thus, models for training individual providers exclusively in medical or graduate school were not included. Similarly, we did not include broad-based team-building interventions focused on entire hospitals or hospital systems unless attendees specifically completed the training together as teams. We included team-building interventions that were delivered under a train-the-trainer model if those trained were then expected to spread the trainings to teams at their home institution.
- Able to be delivered as a specified intervention (e.g. included a workbook, training modules, or workshop components).

### Identifying empirical support (review stage 3)

We conducted a series of additional literature searches in Review Stage 3—one for each team-building intervention identified from reviews in Review Stage 2. The goal of these separate searches was to identify empirical studies evaluating the use of each team-building intervention in non-acute healthcare settings. Sources included Google Scholar, PubMed, associated websites (for team-building interventions that are free and/or publicly available), and direct contact with developers of the team-building interventions. Inclusion criteria for empirical support consisted of the following:

- Inclusion of an intervention based on one of the team-building interventions identified in Review Stage 2 above.
- Inclusion of a systematic evaluation of clinical or staff outcomes in one or more of the four outcome domains described above.

### Reliability

The first author and two co-authors independently rated a subset of ten manuscripts (including reviews, team trainings, and empirical support) identified by the search process above, including some manuscripts that the first author determined had met inclusion criteria, and others that the first author determined had not. Fleiss’s kappa for all three raters for this subset of manuscripts was.70, indicating acceptable reliability [27] for our manuscript identification process.

### Analytic approach

We chose a descriptive approach to achieve our first and second study aims; specifically, we report the characteristics of the team-building interventions and empirical studies identified through our review process. Similar to previous reviews in different healthcare contexts (e.g. [11]) we chose to report the following information for each empirical study: the length of the intervention; the number and types of providers trained; the characteristics of the control condition (if any); whether a pre-training needs analysis was conducted [28]; and whether the intervention was modified from its original version. We also evaluated the quality of the overall body of empirical studies, consistent with criteria on study bias from the Cochrane Collaboration [29]. This involved assessing the risk of selection bias, performance bias, detection bias, attrition bias, and selective reporting in the identified studies.

For our third study goal, the diversity of study designs and outcomes reported in the field made meta-analysis impractical. Instead, we chose to descriptively catalogue the empirical support for each team-building intervention identified in terms of trainee evaluations, teamwork attitudes/knowledge, team functioning, and patient impact. Our approach therefore meets the criteria for a systematic review [30].

## Results

We first describe the results of our multistep search process. We then summarize the characteristics of the team-building interventions and empirical studies. Finally, we present results from empirical studies in our four outcome domains.

### Results from multistep search process

#### Identification of reviews (review stage 1)

**Figure 2:**
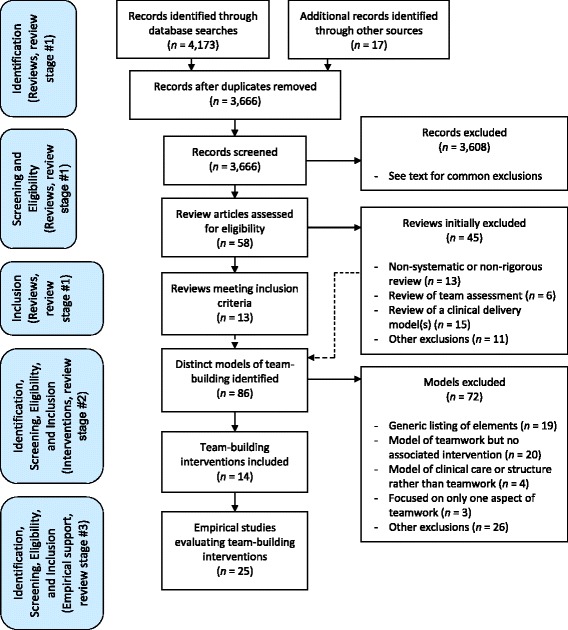
PRISMA Diagram (Modified)

A modified PRISMA diagram (Preferred Reporting Items for Systematic Reviews and Meta-Analyses) can be found in Fig. 2. We screened titles and/or abstracts for 3666 articles identified by our initial search criteria, which endeavored to identify review articles. Consistent with our exclusion criteria, common reasons for exclusion at this stage included: reviews that focused exclusively on acute care teams; reviews that did not specifically address teamwork; reviews of the CCM [26]; reviews focused on principles of team training or education to be applied in graduate or medical school; and reviews of teamwork models that did not include specific team-building interventions. Furthermore, many articles identified at this stage were not in fact review papers; articles that did not meet our definition of a review, but that met criteria for Stages 2 or 3 of our search process as described below, were retained.

This screening resulted in the selection of 58 reviews selected for full-text review, of which 13 met inclusion criteria. Reasons for exclusion at this stage of the review process are detailed in Fig. 2. As described above, however, we also used the remaining 45 reviews to help identify team-building interventions in the next step of our review process.

#### Identification of team-building interventions (review stage 2)

The review articles that we identified in Review Stage 1 above contained references to 86 distinct models of healthcare team-building. A subset of 14 models met criteria for team-building interventions, with common reasons for exclusion also listed in Fig. 2. Table 1 contains brief descriptive information about these team-building interventions, including their delivery format and general content areas.

**Table 1:** Team-Building Interventions for Non-Acute Settings (Alphabetically by First Author)

Team Training	Citation	Description	Empirical Support
TeamSTEPPS	Agency for Healthcare Research and Quality (AHRQ), 2006 [32]	Jointly developed by AHRQ and the Department of Defense, the TeamSTEPPS course consists of a series of modules focusing on team structure, communication, leadership, situation monitoring, mutual support, and other relevant topics. Phase 1 of the traditional TeamSTEPPS curriculum includes a comprehensive needs analysis for participating teams. It was originally developed for crisis or surgical teams, but more recent versions target office-based and long-term care. All modules are available online through the AHRQ website [32]. Also note that Lifewings offers TeamSTEPPS certification programs [60].	One two-part study featuring the long-term care version [55, 63], and five additional studies featuring adaptations of the traditional TeamSTEPPS curriculum for similar outpatient/ambulatory settings [33–35, 46, 54]
CONNECT	Anderson et al., 2012 [64]	“CONNECT is a multi-component intervention that helps staff: learn new strategies to improve day-to-day interactions; establish relationship networks for creative problem solving; and sustain newly acquired interaction behaviors through mentorship” ([64], page 2). It relies on a series of learning sessions and activities conducted in nursing homes over 12 weeks, with an ultimate goal of reducing the incidence of patient falls through improved problem-solving and interaction patterns.	One published study [40], with a larger trial currently underway in 24 facilities
The Arthritis Program - Interprofessional Training Program (TAP-ITP)	Bain, 2014 [53]	TAP-ITP is meant to improve knowledge, skills, and attitudes around interprofessional care. It includes four individual modules that can be delivered in a classroom setting or blended setting (classroom plus online). Support includes learning resources, blogs, discussion boards, and learning portfolios, and it emphasizes an Action-Based Research perspective (with trainees expected to spend time collaborating with one another between modules).	One study [53]
Teams of Interprofessional Staff (TIPS)	Bajnok et al., 2012 [47]	The TIPS training consists of three, 2-day training workshops conducted over 8 months. These workshops include didactics on topics such as developing team culture; conflict resolution; and having difficult conversations. Workshops also involve application of team development strategies, as well as assignment of a mentor/advisor to each team to assist with selection and pursuit of shared team goals.	One study [47]
Team training programme (no formal title provided)	Bunnell et al., 2013 [31]	This program was designed to improve team functioning for outpatient oncology teams using a train-the-trainer model. The 2-hour training session includes general presentation of teamwork principles and supporting evidence, as well as specific interventions related to building teamwork in outpatient oncology settings.	One study [31]
Team training (no formal name provided)	Cashman et al., 2004 [44]	Team training consists of five formal team training workshops conducted over 2-year period, with concurrent increase in regular team meeting times (from 1 h every 4 weeks, to 3 h every 4 weeks). Training topics include stages of group development; personality and work styles; general team-building issues (e.g. related to staffing and turnover); problem-solving; and leadership. Simulations were used to illustrate group processes, and SYMLOG assessment [65] was used to guide discussion.	One study [44]
“3-M” Team Training	Cooley, 1994 [39]	Team training conducted at three workshops (2 h each), conducted 3–4 weeks apart. Workshops included presentations of teamwork concepts, modeling, written practice, role-playing, and analysis of videotaped team meetings. The “3-M” label denotes an organizing framework for the training in “Mapping” skills (to enhance productivity of team meetings); “Mirroring” skills (to enhance communication); and “Mining and refining” skills (to enhance problem-solving capability).	One study [39]
Resource for Education, Audit, and Teamworking (CREATE)	Haycock-Stuart & Houston, 2005 [41]	Team training consists of a series of nine workshops conducted over a 1-year period, oriented around improving primary care teamwork in Scotland. Workshop topics were determined by needs assessment, and included both teamwork-oriented (e.g. communication and planning) and administratively-focused topics (e.g. accreditation issues, appraisal systems, and service redesign).	One study [41]
Expanded Learning and Dedication to Elders in the Region (ELDER)	Lange et al., 2011 [42]	The ELDER project was adapted from the Hartford Foundation’s work [66], and features small-group interactive workshops oriented around interdisciplinary teamworking in the care of older patients. The 3-year project featured approximately 12 educational sessions to be presented to nursing staff in Year 1, an additional six 1-hour sessions to be presented in Year 2, and the additional of simulated patient scenarios in Year 3.	Two studies focused on the implementation of ELDER itself [42, 49], while a third focused on addition of simulation training to the core ELDER curriculum [36]. All three studies were conducted on the same sample.
Training based on the Toronto Framework	Pilon et al., 2015 [20]	The Toronto Framework focuses on three competency domains (Values/Ethics, Communication, Coordination) built over three phases (Exposure, Immersion, Competency). The exposure phase is achieved via a 2-day team retreat, informed by a previously-completed self-assessment. The Immersion phase consists of ongoing team meetings focused on complex case studies; Competency is assessed at repeated team retreats conducted every 6 months.	One study [20]
Interdisciplinary Management Tool (IMT)	Smith et al., 2012 [67]	Developed via research on British intermediate care teams, the IMT is described in detail in a publicly available three-part workbook. Part 1 describes an evidence-based, structured organizational development intervention designed to improve teamwork over a 6-month period with the help of a facilitator. This is ideally accomplished via an initial 1-day workshop and evaluation session, followed by recurring half- to full-day team learning sessions every 2 months (for a total of 3.5 workshop days). Part 2 contains a set of exercises to be completed at the individual and team level, as well as follow-up summaries of relevant research evidence. Part 3 consists of assessment instruments to measure team functioning at the staff and patient levels.	Two studies [52, 68] conducted on same sample
Triad for Optimal Patient Safety (TOPS)	Sehgal et al., 2008 [43]	TOPS involves development of a 4-hour teamwork training program for staff on an inpatient unit combining didactics, facilitated discussion of a safety trigger video, and small-group exercises to enhance communication skills and team behaviors.	Three studies [37, 38, 43] conducted on same sample
Geriatric Interdisciplinary Team Training (GITT)	Siegler, 1998 [66]	The GITT initiative was launched by the John A. Hartford Foundation in 1995, and has continued to inform team-building interventions into the twenty-first century. Programs funded through this initiative were given broad latitude in how specifically to format their team-building interventions, but typically feature a clinical/academic partnership (meaning that some GITT studies have focused on medicine, nursing, or social work studies, while others have focused on intact, enduring clinical teams).	One study focused on intact clinical teams [56], although other studies (e.g. [69]) have presented results for medicine, nursing, and social work trainees (rather than intact clinical teams)
Rehabilitation team training (no formal title provided)	Stevens et al., 2007 [70]	This team training for leaders of rehabilitation teams consists of three phases: “(1) general skills training in team-process (e.g., team effectiveness and problem-solving strategies), (2) informational feedback (e.g., action plans to address team-process problems and a summary of team-functioning characteristics as reported by rehabilitation staff), and (3) telephone and videoconference consultation (e.g., advice on implementation of action plans and facilitation of team-process skills).” The skills training (Phase 1) is conducted in the form of a 2.5-day workshop, and the action plans (Phase 2) provide feedback to participants based on completion of a 67-item pre-training survey. Consultation (Phase 3) consisted of a single group phone or video call conducted 2–3 months post-training. These training activities are all meant to be conducted with team leaders, with the team leaders then working with clinical teams to complete the Phase 2 action plans.	Two studies [45, 70] conducted on same sample

#### Identification of empirical support (review stage 3)

Our search process found 25 empirical studies that presented data on the impact of the 14 identified team-building interventions in non-acute settings. In some cases, the original articles describing the team-building interventions included empirical support that met our inclusion criteria. Table 2 contains brief descriptive information about each of these empirical articles, and the following sections describe characteristics of these studies.

**Table 2:** Empirical Support for Identified Team-Building Interventions

Team-Building Intervention	Citation	Pre-Training Needs Analysis	Topics Covered (beyond Table 1)	Delivery Strategies (beyond Table 1)	Length of Intervention	Number and Types of Providers	Setting	Control Condition
“3-M” Team Training	Cooley, 1994 [39]	No	N/A	N/A	3 months	25 total staff: 11 administrative team members and 14 clinical team members (variety of disciplines including medicine, psychology, social work, physical therapy, and occupational therapy)	Rehabilitation clinic for chronic pain	N/A
CONNECT	Colón-Emeric et al., 2013 [40]	No	Topics covered included: methods for increasing cognitive diversity; developing additional problem-solving skills; developing guidelines for improved interaction patterns	Classroom instruction focused on storytelling, relationship mapping, and feedback. CONNECT includes 4 h of classroom instruction (spread over 2 weeks); completion of individual relationship maps (30 min), and structured mentoring (20 min)	3 months	Intervention: 243 total staff, including primarily nurses and nursing assistants, plus administrators and other staff (specific discipline information collected only for subset who completed surveys)	Intervention: 4 nursing homes, including both VA and non-VA settings	Control teams received FALLS training focused on fall prevention via training modules, teleconferences, and audit/feedback (Intervention group received CONNECT plus FALLS)
						Control: 254 total staff (similar disciplinary makeup as intervention group above)	Control: 4 nursing homes, including both VA and non-VA settings	
CREATE	Haycock-Stuart & Houston, 2005 [41]	Yes	N/A	N/A	1 year	141 total staff: 27 nurses, 31 GP’s, 14 health visitors, 4 practice managers, 31 MSA’s, 34 other staff	Seven general practices (primary care) in one Health Board locality	N/A
ELDER	Lange et al., 2011 [42]	Yes	N/A	N/A	Multi-phase project lasted 3 years total	112 total staff: 53 nurses, 54 nursing assistants, 5 other staff	Four long-term or home care facilities in medically underserved areas	N/A
	Mager et al., 2012 [36]	Yes	Built on Lange et al. [42] by adding simulation of typical clinical cases to ELDER team training	Four simulation sessions lasting an hour, each spaced about a month apart (simulation plus debriefing)	3 months	104 total staff: same population as Lange et al. [42], minus staff from one facility	Same settings as Lange et al.’s study, minus one facility; i.e.., it included two long-term care facilities and one home health care agency	N/A
	Mager & Lange, 2014 [49]	Yes	N/A	N/A	6 months	97 total staff: 42 nurses, 26 nursing assistants; 29 other staff	Five long term or home care agencies in an underserved area of New England	N/A
GITT	Clark et al., 2002 [56]	No	N/A	Specific format for GITT in this study included an initial day-long workshop, followed by 1/2-day follow-up 1 year later (only 3 out of 8 teams participated in the latter as 5 had been disbanded by then)	1 year	94 total staff: 10 physicians, 38 nurses, 16 social workers, 9 administrators, 21 other staff	Eight clinical geriatric teams in various settings: four community hospital/clinics, one nursing home, two mental health agency/centers, one HMO	N/A
IMT	Nancarrow et al., 2012 [52]	Yes	N/A	N/A	6 months	253 total staff: 58 physiotherapists, 56 support workers, 46 occupational therapists, 40 nurses, 53 other staff	Eleven geriatric care teams embedded within home-based care and community care centers	N/A
	Nancarrow et al., 2015 [68]	Yes	N/A	N/A	6 months	Same as Nancarrow et al. [52], minus staff from one team	Same as Nancarrow et al. [52], minus staff from one team	N/A
Rehabilitation Team Training (no formal name provided)	Stevens et al., 2007 [70]	No	N/A	N/A	6 months	29 total staff: 2 team leaders (typically but not exclusively physicians or osteopaths) participated in the training per intervention site, with the understanding that they would spread lessons learned to their teams (1 team sent just 1 leader)	Rehabilitation units emphasizing care for patients with stroke at 15 VA Medical Centers	N/A for this report
	Strasser et al., 2008 [45]	No	N/A	N/A	6 months	Intervention: 227 total staff including many medical disciplines (precise discipline breakdown not reported, but teams included physicians, nurses, occupational therapists, speech-language pathologists, physical therapists, and case managers/social workers)	Same as Stevens et al. [70]. Patients treated: - Intervention: 350 stroke patients, 2337 total patients - Control: 439 stroke patients, 2120 total patients.	Both intervention and control team leaders received team performance profiles and recommendations for how to use this information to improve their team processes.
						Control: 237 total staff (similar disciplinary breakdown as intervention group)		
TAP-ITP	Bain et al., 2014 [53]	No	N/A	N/A	Not reported	22 total staff: 8 physiotherapists, 5 occupational therapists, 9 other clinical staff	Four clinical teams focused on arthritis care in Canada	N/A
Team-STEPPS	Stead et al., 2009 [35]	Yes	N/A	Training delivered via train-the-trainer model	8 months	45 total staff completed assessments: precise discipline breakdown not reported	Clinical team from a mental health site in South Australia	N/A
	Mahoney et al., 2012 [33]	Yes	N/A	Training delivered via train-the-trainer model	8-h train-the-trainer session, remainder of staff to be trained within 45 days	284 full and part time staff, faculty, and admin (188 full or part time clinical including physicians, psychologists, and two 2 nurses; 96 nonclinical staff)	Psychiatric inpatient unit	N/A
	Spiva et al., 2014 [34]	Yes	N/A	Train-the-trainer model, with didactic lecture covering each domain along with video scenarios and debriefing of content covered	9 months	TeamSTEPPS: 18 staff	TeamSTEPPS: 17 bed neurology unit & 16-bed orthopedic unit	No training and continued with usual practice
						Comparison group: 16 staff	Comparison group: 30-bed neurology unit and 22-bed orthopedic unit	
	Treadwell et al., 2015 [46]	Yes	N/A	Hour-long weekly sessions facilitated by case managers; 6 weeks curriculum training, 6 weeks addressing issues of the teams choice	3 months	TeamSTEPPS: 171 total staff including physicians, medical assistants, front desk staff, and others (precise discipline breakdown not reported) Comparison group: 157 total staff	TeamSTEPPS: 25 medical homes Comparison group: 25 medical homes	Curriculum provided by US Department of Health and Human Services: Energize Our Families
	Gaston et al., 2016 [54]	Yes	N/A	2-h training session including didactic instruction along with an audiovisual slide presentation including videos, discussion questions, scenarios, and oncology-specific examples. 10 Master Trainers (MTs) attended a 1-day course, MTs provided coaching on each of the patient care units for the duration	3 months	110 total staff including 92 nurses, 12 Certified Nursing Assistants or healthcare technicians, 6 physicians	3 oncology units	N/A
	Roman et al., 2016 [55, 63]	No	Long-term care version of TeamSTEPPS	Six modules presented in a co-teaching format that encouraged participation and collaboration	One 6-hour training, offered at multiple times from Sept-Dec 2015	41 staff including managers, nurses, nursing assistants, social worker, therapists, administrative staff, and others (precise discipline breakdown not reported)	Long-term care facility	N/A
Team training (no formal name provided)	Cashman et al., 2004 [44]	No	N/A	N/A	2 years	6 total staff: 1 each of physician, nurse practitioner, physician assistant, registered nurse, health assistant, and outreach worker/case manager	Primary care team in one New England community health center	N/A
Team training programme (no formal title provided)	Bunnell et al., 2013 [31]	Yes	N/A	N/A	2-h session (delivered once)	104 total staff: 20 physicians, 47 nurses, 4 pharmacists, and 35 support staff (trained in sets of about 20 staff each)	Outpatient breast cancer treatment center	N/A
TIPS	Bajnok et al., 2012 [47]	No	N/A	N/A	8 months	32 total staff: 5 physicians, 10 nurses, 6 physical or occupational therapists, 11 other staff	Five healthcare teams from Ontario: included four non-acute care clinics and one emergency department	N/A
TOPS	Sehgal et al., 2008 [43]	No	N/A	N/A	½ day (delivered six times to cover all partici-pants)	225 total staff: hospitalists, nurses, pharmacists, internal medicine residents, and other staff (precise numbers from each discipline not reported)	Inpatient medical unit at an academic medical center	N/A
	Blegen et al., 2010 [38]	No	In addition to core TOPS intervention, patient goals were also solicited unit-wide and posted in patient rooms to facilitate communication	In addition to core TOPS intervention, educational sessions were run by Triad Unit Safety Teams (TrUSTs) to emphasize TOPS lessons	Not reported	454 total staff: 182 nurses, 102 medical residents, 53 pharmacists, 43 attending physicians, 54 other staff	Study sample included same inpatient unit as Sehgal et al. [43], plus two additional inpatient units from other medical centers	N/A
	Auerbach et al., 2011 [37]	No	Same as Blegen et al. [38]	Same as Blegen et al. [38]	Same as Blegen et al. [38]	Same as Blegen et al. [38]	Same as Blegen et al. [38]	N/A
Toronto Framework	Pilon et al., 2015 [20]	No	N/A	N/A	2 years (although designed to be ongoing)	6 total staff: 2 nurses, 1 pharmacist, 1 social worker, 1 physician, 1 physician assistant	Primary care setting associated with Vanderbilt school of nursing, serving low-income/ disadvantaged patients	N/A

### Characteristics of team-building interventions and empirical studies

#### Content and format of team-building interventions

As described in Table 1, nine of 14 team-building interventions (64%) were built around one or more formal workshops, Additionally, eight of the 14 team-building interventions (57%) explicitly featured ongoing learning activities that were embedded into periodic team meetings or available online. A total of nine of the 14 team-building interventions (64%) explicitly described the inclusion of role-plays, interactive discussions, simulation, or other ways to actively engage participants in addition to more traditional didactics. One intervention [31] was designed to be disseminated via a train-the-trainer model, and one additional team-building intervention (TeamSTEPPS [32]) was delivered via a train-the-trainer model in three of the empirical studies validating it [33–35].

As demonstrated in Table 2, 12 of 25 empirical studies (48%) included a pre-training needs analysis specifically with the teams to be trained. Of the studies featuring a needs analysis, about half were studies of TeamSTEPPS [32], which explicitly includes a training needs analysis as part of its Phase 1.

Furthermore, three empirical articles clearly described making modifications to the team-building intervention in question. These modifications took the form of additional simulation modules [36] or mechanisms for soliciting patient goals [37, 38].

#### Length of team-building interventions identified

As described in Table 2, the team-building interventions evaluated in empirical studies ranged from single-day sessions (or portions thereof) to multi-year initiatives. The median length of team-building interventions was 6 months among the 18 empirical articles that reported such data. For the remaining seven empirical articles it was impossible to tell how long the intervention truly lasted, either because the total length was nort reported or because the interventions described therein followed a train-the-trainer model in which team leaders were expected to spread lessons to their individual teams (e.g. [33])

#### Settings in which studies were conducted

As shown in Table 2, empirical studies were conducted in a variety of non-acute settings including three studies in rehabilitation clinics (e.g. [39]), two studies in nursing homes (e.g. [40]), three studies in primary care (e.g. [41]), five studies in long-term care facilities (e.g. [42]), and seven studies in community care or other outpatient settings (e.g. [31]). Four studies were conducted in inpatient units [33, 37, 38, 43], but (consistent with our review criteria) were included if the team-building interventions in question focused on teamwork outside of crisis situations such as cardiac arrests.

#### Numbers and types of providers trained

The numbers and types of providers trained varied considerably, consistent with the variety of settings in which the empirical studies included in this review took place. Among the 14 studies that reported a specific disciplinary breakdown, enrolled staff included 679 nurses (38% of participants), 373 physicians (21%), 92 nursing assistants (5%), 87 support staff (5%), 9 administrators (< 1%), and 556 other staff (31%). The number of providers trained ranged from the single digits (e.g. for pilot studies with one small team [44]) to over 400 (e.g. for studies involving clinical and non-clinical staff from multiple clinics [38]). The median number of staff included in these studies was about 100, with the caveat that some studies used a train-the-trainer model (in which cases the total number of staff affected by the training would be higher than what was reported in the article).

#### Characteristics of the control conditions

As Table 2 reveals, very few empirical studies included a control condition. Two studies included comparisons to other teams that had received no intervention [25, 34], while two additional studies had designs in which both the intervention and control teams received some shared components, and one team also received the team-building intervention in question [40, 45]. In only one case [46] did the control team receive another active intervention that was distinct from the training received by the intervention group.

#### Quality of empirical studies

Data from Table 2 suggest that many of the empirical studies we identified should be considered at high risk of the five types of bias specified by the Cochrane Collaboration [29]. There was marked potential for selection bias in at least 23 of 25 studies, given that only two studies appeared to include credible control conditions and the fact that teams were typically not chosen at random to participate in the empirical studies. Similarly, performance bias and detection bias—which can occur when either participants or raters, respectively, are unblinded—were nearly ubiquitous among empirical studies given that blinding was typically difficult (when control conditions were clearly differentiable from intervention conditions to participants) or impossible (when no control condition was included). Furthermore, most outcome assessments (e.g. trainee evaluations, team attitude/knowledge checks, and team functioning assessments) were completed by trainees themselves rather than independent observers. In fact, only four studies included assessments of team attitudes/knowledge or team functioning derived from observer ratings [31, 36, 37, 44]. Attrition bias was evident, as several studies had teams drop out prior to post-intervention data collection. Finally, selective reporting bias was likely as many studies did not describe which of their outcome measures was considered primary, focused on specific sub-domains without explaining why those subdomains were selected, or highlighted results from only a subset of teams studied.

### Outcomes in four domains

Table 3 contains results regarding the four outcome domains. With some exceptions, the 12 empirical studies that collected trainee evaluations reported positive scores among participating staff in this domain (with 68–100% rating their experiences as positive). Only one of the six studies that assessed teamwork attitudes/knowledge [47] found statistically significant improvement in knowledge of teamwork principles as evidenced by the *Outcomes* elements of the WeLearn framework [48]. Qualitative results from that study also supported increased awareness of teamwork principles. Other studies, however, found no statistically significant differences in attitudes toward teamwork pre- to post-intervention [35, 49], or between the intervention and control group [34].

**Table 3:** Outcomes for Identified Team-Building Interventions

Team-Building Intervention	Citation	Outcomes
“3-M” Team Training	Cooley, 1994 [39]	Trainee Evaluations: Average ratings for each of the three workshops ranged from 3.94 to 4.35 on a 1–5 Likert scale (standard deviations not reported). Participants found workshop sessions generally well-organized and useful, but would have appreciated more time to develop skills.
		Team Functioning: Results for each conceptual domain targeted by the training (mapping, mirroring, and mining/refining) showed improvement that did not reach statistical significance.
CONNECT	Colón-Emeric, 2013 [40]	Team Functioning: Significantly improved communication and safety culture across intervention and control; trend-level findings of greater communication improvement for intervention than control (*p = .*06)
		Patient Impact: Exploratory findings suggested a greater decrease in the number of falls in intervention nursing homes compared to control nursing homes (not statistically significant)
CREATE	Haycock-Stuart & Houston 2005 [41]	Trainee Evaluations: 69% thought CREATE was relevant; 80% said it met some of their educational needs (clinical staff appreciated it more than administrative staff); 68% wanted it to continue.
		Team Functioning: Self-reports post-intervention suggested improved communication and the development of formalized meetings in at least one practice; additional analyses suggested statistically significant improvement in several self-reported teamwork variables (e.g. clear objectives, evaluating success in meeting practice objectives, meeting attendance, communication)
ELDER	Lange et al., 2011 [42]	Trainee Evaluations: Generally positive but not subjected to empirical testing
	Mager et al., 2012 [36]	Trainee Evaluations: 97–100% of staff at each site rated the training positively
		Teamwork Attitudes/Knowledge: Notes and checklists indicated good communication, respect, and collaboration during the simulations themselves (although not subjected to pre-post analysis)
	Mager and Lange, 2014 [49]	Trainee Evaluations: Qualitatively, participants reported preferring innovative teaching methods (e.g. case-based discussion) over traditional lecture
		Teamwork Attitudes/Knowledge: Participants did not show statistically significant improvement in knowledge of team concepts (based on a GITT instrument) or scores on an Interdisciplinary Teamwork IQ assessment
GITT	Clark et al., 2002 [56]	Team Functioning: No statistically significant changes for domains such as communication and cohesion (based on a team function assessment scale)
IMT	Nancarrow et al., 2012 [52]	Trainee Evaluations: Generally positive, but some participants expressed concerns about the amount of time required to attend workshops and complete associated assessments
		Team Functioning: Workforce Dynamics Questionnaire [51] suggested improved team working score improved over time (*p-*value significant but not reported); no statistically significant change in several other teamwork domains; qualitative assessment (*n* = 15) suggested overall improved teamwork
		Patient Impact: Changes in patient satisfaction pre- to post- intervention significant at some but not all sites
	Nancarrow et al., 2015 [68]	Trainee Evaluations: This study expands on the findings from the trainee evaluations and qualitative findings reported in the Nancarrow et al. [52] (with results being generally but not universally positive)
Rehabilitation Team Training (no formal name provided)	Stevens et al., 2007 [70]	Trainee Evaluations: 100% of attendees agreed or strongly agreed that workshop met goals of emphasizing team functioning and its impact on patient outcomes; attendees less enthusiastic about written information summarizing survey responses related to team functioning
	Strasser et al., 2008 [45]	Patient Impact: More patients treated by intervention teams gained above the median in motor function from Functional Independence Measure (FIM [71]); difference in increase: 13.6%; *p* = 0.03; no difference in length of stay or community discharge
TAP-ITP	Bain et al., 2014 [53]	Trainee Evaluations: W(e)Learn Program Evaluation Survey [48] indicated general satisfaction with the program
		Team Functioning: Self-reports of collaboration, cohesion, communication, and conflict resolution improved post-intervention and at 1-year follow-up on the Bruyère Clinical Team Self-Assessment on Interprofessional Practice [72]
TeamSTEPPS	Stead et al., 2009 [35]	Trainee Evaluations: Evaluations were generally positive for participating staff, but specific results were neither reported nor subjected to statistical testing
		Teamwork Attitudes/Knowledge: Some improvements were reported in teamwork-related knowledge, skills, and attitudes, but overall change scores were not statistically significant
		Team Functioning: Statistically significant improvement in communication (*p* < .05) from pre- to post-intervention
		Patient Impact: Reduced seclusion rates (*p* < .001) from pre- to post-intervention
	Mahoney et al., 2012 [33]	Team Functioning: Significant increases in Teamwork Attitudes Questionnaire [73] from pre-intervention to 1-year follow-up (*p* < .01 for five of seven subdomains)
TeamSTEPPS (continued)	Spiva et al., 2014 [34]	Teamwork Attitudes/Knowledge: Compared to the control group, the intervention group did not experience statistically greater improvement on TeamSTEPPS Teamwork Attitudes measure
		Team Functioning: Compared to the control group, the intervention group did not experience statistically greater improvement on the Hospital Survey on Patient Safety Culture (HSOPSC [74]) subdomains; similarly, no statistically greater improvement on TeamSTEPPS Team Members’ Perceptions of Team Effectiveness
		Patient Impact: Intervention group fall rates reduced by 62% and injury rates by 71% (compared to increased rates for control group)
	Treadwell et al., 2015 [46]	Team Functioning: Intervention group had significantly higher ratings of team collaboration post-intervention than did the comparison group (*p* < .001)
	Gaston et al., 2016 [54]	Trainee Evaluations: Training rated as “good” to “excellent” by 96–100% of participants
		Team Functioning: Teamwork Perceptions Questionnaire [75] and HSOPSC [74] subscale scores showed statistically significant improvement from pre- to post-intervention (*p* < .001)
	Roman et al., 2016 [55, 63]	Teamwork Attitudes/Knowledge: Participants endorsed increased awareness of the need for open communication (not subjected to statistical testing)
		Team Functioning: Statistically significant improvement from pre- to post-intervention in all five teamwork-related subscales assessed (all *p* < .01 from custom measure)
Team Training (no formal name provided	Cashman et al., 2004 [44]	Team Functioning: Post-intervention SYMLOG (Systematic Multiple Level Observation of Groups [76]) showed significant improvements in task orientation (i.e. feeling sense of shared goals/tasks), friendliness, and dominance (i.e. comfortable being assertive in working toward shared goals), but findings were not evaluated via statistical testing. One-year follow-up showed regression for some of these measures (apparently based on frustration at slow rate of change and bureaucratic restrictions)
Team Training Programme (no formal title provided)	Bunnell et al., 2013 [31]	Team Functioning: Staff consistently reported post-intervention improvements in team-related clinical care processes, although this was not subject to statistical testing; missing orders for unlinked visits dropped significantly post-intervention (30 to 2%, *p* < .001); no statistically significant change in uncommunicated order changes pre- to post-intervention
TIPS	Bajnok et al., 2012 [47]	Trainee Evaluations: Generally positive, especially related to setting shared team goals, but results were not subject to statistical tests
		Teamwork Attitudes/Knowledge: Quantitative pre-post surveys showed statistically significant improvements in W(e)Learn [48] constructs of content, service, and outcomes
		Team Functioning: Surveys suggested improved team functioning but not subjected to statistical tests
		Patient Impact: Provider surveys suggested improved clinical outcomes but not subjected to statistical tests
TOPS	Sehgal et al., 2008 [43]	Trainee Evaluations: Almost universally positive, with 99% of attendees reporting that they would recommend the training to their peers; mean overall rating of the training was 4.5 (sd = 0.79) on 1–5 Likert scale (but not subjected to statistical tests)
	Blegen et al., 2009 [38]	Team Functioning: Within-unit teamwork HSOPSC [74] showed no statistically significant change from pre- to post-intervention (statistically significant findings emerged only when one site was dropped from the analyses)
	Auerbach et al., 2011 [37]	Team Functioning: Patients were significantly more likely to report good team functioning on the part of their clinicians post-intervention
		Patient Impact: No statistically significant effects on readmission or length of stay; patients were more likely post-intervention (at the trend level) to indicate that their providers had made a mistake that affected their care
Toronto Framework	Pilon et al., 2015 [20]	Team Functioning: No change in TDM [50] scores over 2 years

Eighteen empirical studies also reported results from post-training assessments of team functioning such as the Team Development Measure (TDM [50]) or Workforce Dynamics Questionnaire [51]. Most such studies showed improvement in a few [35, 40, 46, 52] or several teamwork-related domains [33, 41, 53–55]. However, other studies did not find statistically significant improvement in team functioning post-intervention [20, 34, 38, 39, 56]. This variability also manifested within specific teamwork domains (e.g. some studies reported enhanced communication as a result of the team-building intervention [40, 41, 53] while others did not [39, 44]).

Additionally, six studies investigated clinical outcomes or patient satisfaction for patients treated by clinicians who had participated in the team-building intervention, and, of the studies that did investigate these outcomes, findings were generally mixed. For example, two studies that investigated falls in nursing homes [40] and an orthopedic unit [34] found at least a modest reduction in falls from pre- to post-intervention, but other studies either found no statistically significant changes in clinical outcomes (e.g. [37]) or did not subject such outcomes to statistical testing (e.g. [47]).

## Discussion

To our knowledge, this is the first systematic review of healthcare teamwork to focus specifically on the empirical support for team-building interventions for providers in non-acute treatment settings such as primary care or rehabilitation clinics. We only found 14 distinct team-building interventions that met our criteria, which is a striking contrast to the large number of such interventions [11, 57] that have been applied in acute care or emergency settings. Furthermore, several factors (including a heterogeneity of outcome measures, paucity of control conditions, and small number of studies evaluating each team-building intervention) complicated the interpretation of results, making it difficult to determine which of the team-building interventions we identified would be expected to outperform the others. Nonetheless, we hope that our analyses prove useful for outpatient clinic administrators and managers interested in boosting the effectiveness of their clinical teams.

### Outcomes of team-building interventions

Consistent with our guiding conceptual model (Team Effectiveness Pyramid; Fig. 1), we reviewed outcomes in four domains: trainee evaluations; attitudes toward, and knowledge about, teamwork; team functioning; and patient impact. As detailed in Table 3, empirical studies generally reported positive trainee evaluations, although a shortage of credible comparison conditions (e.g. different team-building approaches) made it difficult to determine how meaningful this finding is. Some of the studies we reviewed also found their interventions to be associated with improvements in knowledge of the principles of team-based care or attitudes toward the importance of teamwork—but only one study found such improvements to achieve statistical significance, and several studies either found no significant change in this domain or minimal differences between the intervention and control teams.

Similarly mixed results emerged for team functioning, with some studies finding robust improvements associated with team-building interventions. Others found significant changes for only a small set of team functioning variables, or no differences at all associated with the team-building intervention. In several cases, positive results appeared to be selectively chosen from among many potential subdomains (e.g. focusing on positive findings for one aspect of communication, while downplaying negative findings for other aspects of communication).

Fewer studies investigated clinical outcomes or patient satisfaction for patients treated by clinicians who had participated in team-building. The existing findings in these domains were generally mixed, although two studies that investigated falls in nursing homes [40] and an orthopedic unit [34] respectively found at least a modest reduction in falls from pre- to post-intervention. Clearly, future research should include assessments of patient impact to fully identify the potential benefits of such interventions.

### Characteristics of team-building interventions

As described in Table 1, many team-building interventions featured a workshop as a central component, with of subset of these including either repeated workshops or use of ongoing (e.g. weekly) team meetings to continue developing teamwork practices. Based on this variation, it is not surprising that the total length of the team trainings ranged widely; median length was about 6 months of active teamwork development. Workshop activities ranged from traditional classroom instruction, to team-building exercises, to case-based learning. In at least one case, trainee evaluations revealed that participants preferred more innovative teaching methods (e.g. case-based discussion [49]), a finding that is consistent with previous reviews [58] and adult learning theory [59].

The teamwork topics included in this review’s team-building interventions were similar to those identified in previous reviews from acute healthcare settings (e.g. [11]). These topics most commonly included communication, leadership, problem-solving, conflict management, and team goal-setting. Many of the team-building interventions we identified also included some clinical training or coverage of administrative issues such as accreditation (e.g. [41]). A pre-training needs analysis is a common component of team-building [11]; 48% of our identified empirical articles (12/25) noted inclusion of such a needs analysis. Our guiding conceptual model (Team Effectiveness Pyramid; Fig. 1) included the capacity for engaging in process improvement as a possible result of enhanced teamwork, but this was rarely mentioned in the identified team-building interventions.

Among the team-building interventions identified via this review, TeamSTEPPS (Team Strategies and Tools to Enhance Performance and Patient Safety) was the only one that has been tested by more than one research team in more than one sample. The core TeamSTEPPS curriculum was initially designed for acute care settings by the Agency for Healthcare Research and Quality (AHRQ [32]), but individual research teams (e.g. [34, 46]) and AHRQ itself have successfully adapted TeamSTEPPS for use in non-acute settings. Strengths of the TeamSTEPPS approach include the ready availability of supporting materials from AHRQ website [32], as well as the incorporation of a pre-training needs analysis to ensure that the specific curriculum implemented matches the needs of the team being trained. Furthermore, for organizations desiring more explicit support in implementing TeamSTEPPS beyond the materials provided by AHRQ, several private entities offer TeamSTEPPS-oriented trainings (e.g. Lifewings [60]).

### Characteristics of empirical studies

With the exception of TeamSTEPPS (described above), most of the interventions we identified have only been validated in one study—which was typically published by the developers of the intervention itself. Furthermore, in several cases, multiple empirical studies were published on the same validation sample, and the typical empirical study was conducted with just 6–8 teams within six clinics (for a total of about 100 staff trained in the median study we reviewed). These numbers suggest that few team-building interventions (beyond TeamSTEPPS) have been subjected to exhaustive empirical study in the form of multiple studies conducted by different research teams across multiple non-acute samples.

It was difficult to determine whether individual research teams made systematic modifications to the team-building interventions in the empirical articles we identified. Many such interventions are inherently flexible, making it nearly impossible to differentiate training-consistent from training-inconsistent adaptations based on published literature. However, three articles clearly described either the addition of simulation modules [36] or solicitation of patient goals [37, 38]. Following the conventions of Stirman and colleagues [61], these represent modifying the intervention format as well as adding elements to the training content.

Our study results indicate a high risk of bias in several domains specified by the Cochrane Collaboration [29]. These include selection bias, performance bias, detection bias, attrition bias, and selective reporting bias. The first three of these potential biases were difficult to avoid given the paucity of control conditions (Table 2). Furthermore, the few control conditions we found typically involved the commitment of fewer resources than the interventions being studied. Close inspection of Table 2 reveals that several studies were prone to attrition bias (based on teams dropping from the study before final data collection) or selective reporting bias (by emphasizing significant results and downplaying equivocal results even within teamwork domains). Thus, none of the studies we found could definitively address the question of whether the resources put into team-building would have been better spent on more clinical or administrative staff for the team(s) in question.

### Implications for future research and practice

Taken together, our results emphasize that research on team-building in non-acute healthcare settings lags behind that in acute settings. Furthermore, we did not find consistent positive results—in terms of improvement in teamwork attitudes/knowledge, team functioning, or clinical outcomes—across the studies we reviewed. Thus, an important next step for the field is to determine *the circumstances under which team-building will be most effective in non-acute healthcare settings*. For example, teams in some settings may have limited overlap in caseloads among team members, making it especially difficult to establish shared goals within the team or foster enthusiasm for team-building. We have seen this dynamic occur in outpatient mental health teams [62], as staff may find it difficult to commit to team meetings and shared activities if only a small portion of their caseload is treated by other members of the team. In such situations, it may be important to better align team caseloads to maximize the potential for coordination within the team before team-building can begin in earnest.

Similarly, more research is needed regarding what we have labeled foundational resources (Level 1 of our Team Effectiveness Pyramid, Fig. 1). There is broad agreement that such resources are required for teamwork to blossom—and well-developed bodies of literature on the importance of the individual foundational elements listed in the Pyramid (e.g. [23]). However, we are not aware of any concrete methods for determining whether such resources are sufficiently in place for team-building to be indicated. For example, if an outpatient clinic is extremely short-staffed, then taking clinical time offline for team-building may simply result in more stress and burnout on the part of clinic staff. In such circumstances, it is possible that team-building should be postponed until new staff can be hired or patient flow within the clinic can be adjusted to reduce provider burden. Consistent with this, survey results from one empirical study we reviewed indicated that providers were concerned about the amount of time required for team-building [52]. A robust method for determining the minimum levels of foundational resources needed for non-acute healthcare teams to profitably engage in team-building would be valuable contribution to the field. In the meantime, we recommend that any team-building intervention include a thorough pre-training needs analysis [28] that includes an assessment of the resources available to the team. Ideally, such analysis would inform possible adaptations of the team-building intervention itself to match local needs.

### Limitations

Results from this review should be considered in light of several limitations. First, given the breadth of the field, we relied on a multi-stage search process—identifying reviews, then using these reviews to identify team-building interventions, and finally using those reports to identify articles that empirically evaluated each intervention. This leaves the possibility that we missed interventions or studies that have not been included in previous reviews—especially team-building interventions developed too recently to be included in published reviews. However, this type of method has been used before in different healthcare contexts [25], and the use of not just a literature search but also the examination of the reference lists of dozens of review papers leaves us confident in the scope of team-building interventions we identified. Furthermore, our Review Stage 1 resulted in the identification of several team-building interventions directly. We also contacted intervention developers directly to inquire about potential other empirical reports we might have missed; in no cases did this reveal studies that our search process did not. Second, we were limited by the amount of information available in the peer-reviewed articles we reviewed. It is therefore possible that we may have underestimated the extent to which certain elements (such as a pre-training needs analysis or systematic modifications) were used in our reviewed studies. However, we would not expect this to affect our core study findings regarding the outcomes of team-building interventions. Third, given the diversity in the literature, we were unable to conduct a formal meta-analysis. Instead, we endeavored to narratively describe results in four outcome domains that are prominent within the literature and aligned with our Team Effectiveness Pyramid conceptual model (Fig. 1).

## Conclusions

To our knowledge this is the first review of team-building interventions to focus specifically on non-acute healthcare settings. This evidence base is much less developed than the parallel literature for emergency rooms, surgical departments, and other crisis-oriented settings. Of the interventions identified in our systematic review, only TeamSTEPPS [32] has been tested in multiple non-acute settings by distinct research teams. While results for most of the studies included in this review were generally positive, these findings are tempered by a lack of control conditions, inconsistency in outcome measures, and high probability of bias [29]. Furthermore, the fact that the majority of team-building interventions have only been tested in one study made it impossible to confidently compare results across different interventions and settings.

In conclusion, there is tentative evidence that robust team-building interventions can be helpful in improving team functioning and result in positive patient impacts in non-acute healthcare settings, but this evidence base lags far behind that for acute settings. Considering this uncertainty alongside the well-recognized costs of poor healthcare teamwork [7] underscores the critical need for additional research to determine the best ways to enhance teamwork in these settings, the circumstances under which certain interventions may be more effective than others, and rigorous and consistent ways to measure the impact of such interventions.

## Abbreviations

AHRQ: Agency for Healthcare Research and Quality
CCM: Collaborative Care Model
CREATE: Resource for Education, Audit and Teamworking
CRM: Crew Resource Management or Crisis Resource Management
ELDER: Expanded Learning and Dedication to Elders in the Region
FIM: Functional Independence Measure
GITT: Geriatric Interdisciplinary Team Training
HSOPSC: Hospital Survey on Patient Safety Culture
IMT: Interdisciplinary Management Tool
PRISMA: Preferred Reporting Items for Systematic Reviews and Meta-Analyses
SYMLOG: Systematic Multiple Level Observation of Groups
TAP-ITP: The Arthritis Program - Interprofessional Training Program
TDM: Team Development Measure
TeamSTEPPS: Team Strategies and Tools to Enhance Performance and Patient Safety
TIPS: Teams of Interprofessional Staff
TOPS: Triad for Optimal Patient Safety
